# Complex foot deformities associated with lower limb deformities: a new therapeutic strategy for simultaneous correction using Ilizarov procedure together with osteotomy and soft tissue release

**DOI:** 10.1186/s13018-020-02021-w

**Published:** 2020-10-23

**Authors:** Qin Boquan, Ren Yi, Gan Tingjiang, Liu Xi, Zhang Hui

**Affiliations:** 1 Department of Orthopaedic Surgery, West China Hospital, Sichuan University, No. 37, Guoxue Avenue, Chengdu, 610041 Sichuan Province China; 2 Disaster Medicine Center, Sichuan University, Chengdu, 610041 Sichuan Province China

**Keywords:** Ilizarov, Foot deformity, Lower limb deformity, Soft tissue release, Osteotomy

## Abstract

**Aim:**

The aim of the current study is to introduce a new therapeutic strategy for simultaneous correction of complex foot deformities (CFD) and the associated lower limb deformities (LLD) by using Ilizarov technique with osteotomy and soft tissue procedure and to report its early clinical results.

**Methods:**

A retrospective review of CFD associated with LLD simultaneous correction utilizing the Ilizarov procedure together with osteotomy and soft tissue balance from 2015 to 2019 was conducted.

**Results:**

Thirty-two patients were followed for an average of 42.8 months. The mean external fixation time (EFT) was 6.5 months. The mean healing index (HI) was 1.7 months/cm. At the time of fixator removal, plantigrade feet were achieved in all patient and lower limb deformities were corrected. No recurrence of the deformities occurred. The mean LLRS AIM score was improved from 7.5 to 0.3. At the final follow-up, the ASAMI-Paley score was graded as excellent in all limbs in the aspect of bone results, and functional results were defined as excellent in 29 (90.6%) limbs and good in 3 (9.4%) limbs. The mean modified Dimeglio score was significantly improved from 7.2 to 1.3. No deep infection of the osteotomy site or nonunion was noted in the current study.

**Conclusion:**

The therapeutic strategy by using the Ilizarov procedure together with osteotomy and soft tissue balance is a safe and effective way to simultaneously correct CFD and LLD.

**Level of evidence:**

Level IV, retrospective case series

## Introduction

Complex foot deformities (CFD) can be defined as multiplanar deformities, which are commonly complicated with lower limb deformities (LLD) [1]. The correction of this kind of deformities continues to be a difficult surgical challenge all over the world [2, 3]. Such deformities can be congenital or acquired, which are caused by neuromuscular disease, congenital talipes equinovarus (CTEV), neglected or relapsed clubfoot, poliomyelitis, osteomyelitis, bum contracture, and so on.

A large number of studies reporting various efforts in regard of surgical correction, including soft tissue release, muscular balance [4, 5], various osteotomies [6–8], or arthrodesi s[9], have been published in the past decades. However, the complex foot deformity does not only influence the foot and ankle region alone. It is often complicated with simultaneous deformity of the lower limb, including limb length discrepancy, angulation, and rotatio n[10]. Such complicated lower limb deformity can further deteriorate the patient’s walking disorder. The resultant abnormal alignment may lead to accelerated degeneration of the joints both in the affected limb and in the limb on the opposite side, causing severe chronic pain and disability [11]. With time passes by, the degeneration may involve the vertebral column. In the late stage, the patient may even have difficulty to stand [12]. Yet the interest of most of the previous studies has been concentrated on the complex foot deformity itself, while ignoring the associated lower limb deformities. Nevertheless, the correct alignment of the foot and ankle region can only be established based on the correct alignment of the ipsilateral lower limb. Furthermore, the lower limb deformity itself as well as the pain and dysfunction that result from joint degeneration may continue to impair the patient’s walking ability even after the CFD has been successfully corrected [13]. Therefore, the treatment of the associated LLD is actually no less important than the treatment of CFD itself. However, the correction of CFD and LLD has some cross impact on each other. On the one hand, if the CFD is chosen to be treated first, it will be hard for the surgeon to determine the correct alignment of the foot while the limb is still misaligned. On the other hand, if the LLD is corrected first, the CFD may still interfere with weight-bearing after the surgery, resulting in disuse osteoporosis, delayed ossification, nonunion, and instrument failure, during the Ilizarov procedure for the correction of LLD. In the current study, a therapeutic strategy for simultaneous correction of CFD and LLD by using necessary osteotomy and soft tissue procedure in the affected foot together with the Ilizarov technique has been introduced. The early clinical result of this technique is reported.

## Patients and methods

Inclusion criteria include (1) patients’ age ≥ 18 years or skeletal mature, (2) CFD combined with LLD, (3) the deformities were complex and rigid, (4) Dimeglio scores ≥ 6, and (5) LLRS AIM scores ≥ 3.

Exclusion criteria include (1) lost follow-up and (2) incomplete medical records.

According to the criteria above, 32 patients (21 males,11 females) who had CFD combined with LLD were selected into our study. Its duration ranged from 3 to 43 years (mean was 13.7 years). All the patients were older than 18 years (age ranged from 18 to 58 years, with a mean of 33.6).

Thirteen out of 32 patients were students, eight were workers, two were employees, two were doctors, and seven patients had no job.

The etiologic factors were poliomyelitis (10 patients), congenital talipes equinovarus (CTEV) (6 patients), osteomyelitis (2 patients), burn contractures (2 patients), traumatic injuries (4 patients) and neglected, relapsed clubfoot ( 6 patients), or tethered cord syndrome (TCS) (2 patients).

The patient’s demographics are shown in Table 1.

**Table 1:** Patient demographics

	n	%	Range	Mean	Total
Age			18–58	33.6	32
Sex					32
Male	21	66%			
Female	11	34%			
Laterality					32
Left	14	44%			
Right	16	50%			
Bilateral	2	6%			
Etiology					32
Poliomyelitis	10	31%			
CTEV	6	19%			
Osteomyelitis	2	6%			
Burn	2	6%			
Trauma	4	13%			
Clubfoot	6	19%			
TCS	2	6%			
Foot					
Equinus	6	19%			
Deformities					
PEV	13	41%			
Pesplanovalgus	5	16%			
Cavovarus	8	25%			
Duration			3–43	13.7	
Follow up			33–62	42.8	
EFT			5.5–8.0	6.5	
HI			1.0–3.2	1.7	

*CTEV* Congenital talipes equinovarus, *PEV* Pes equinovarus, *EFT* External fixation time, *HI* Healing index, *TCS* Tethered cord syndrome

### Preoperative examination

**Fig. 1:**
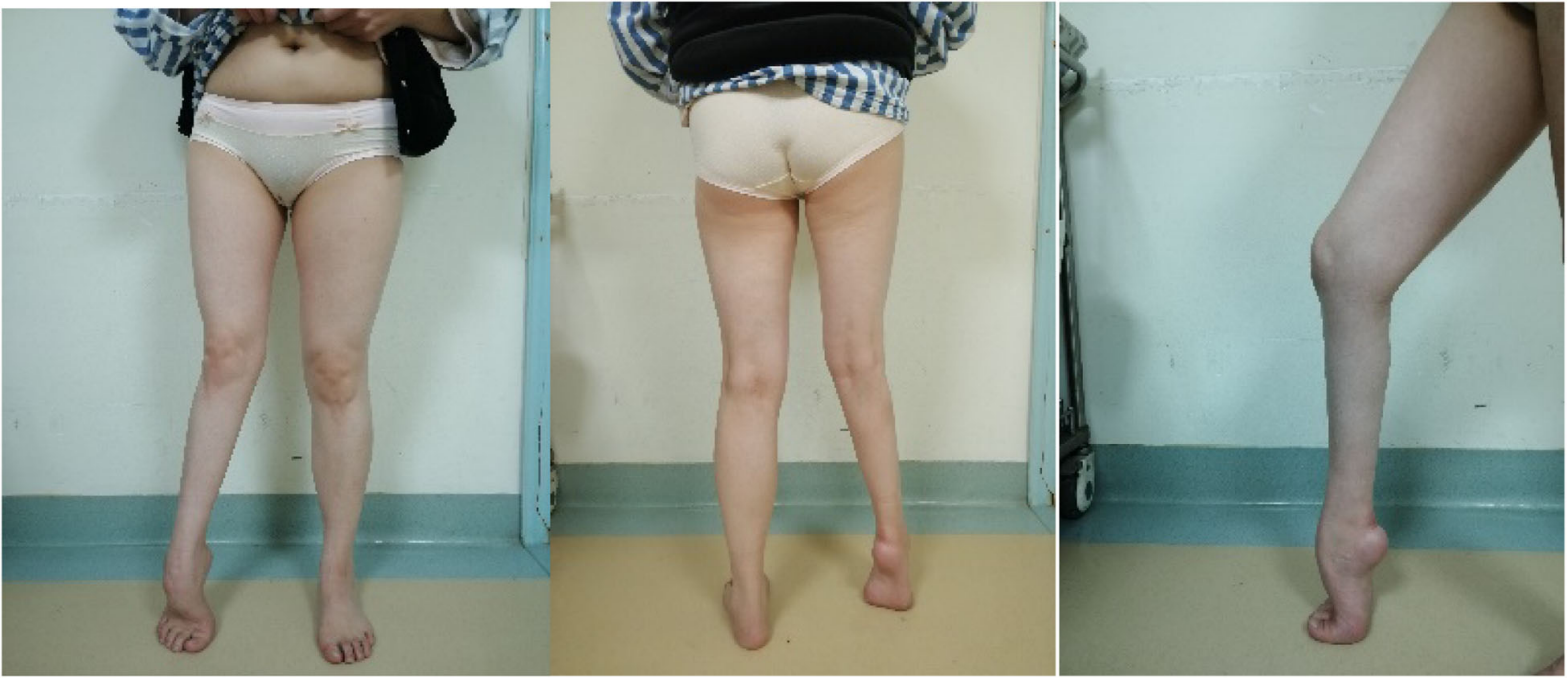
Female, 46 years, who was diagnosed poliomyelitis. Preoperative standing appearance of the patient

All patients received preoperative detailed clinical examination and imaging examination of both lower limbs, foot, and ankle (Fig. 1). A thorough history and examination often establishes the cause of deformity. Clinical examination should evaluate the components of any deformity; the length of both lower limbs; the gait circle; forefoot-hindfoot relationships (e.g., the Coleman block test [14], peek-a-boo sign); hip, knee, ankle, subtalar, midtarsal, and toe joints ranges of movement; lower limb and foot muscle strength; and the condition of the skin and soft tissues.

**Fig. 2:**
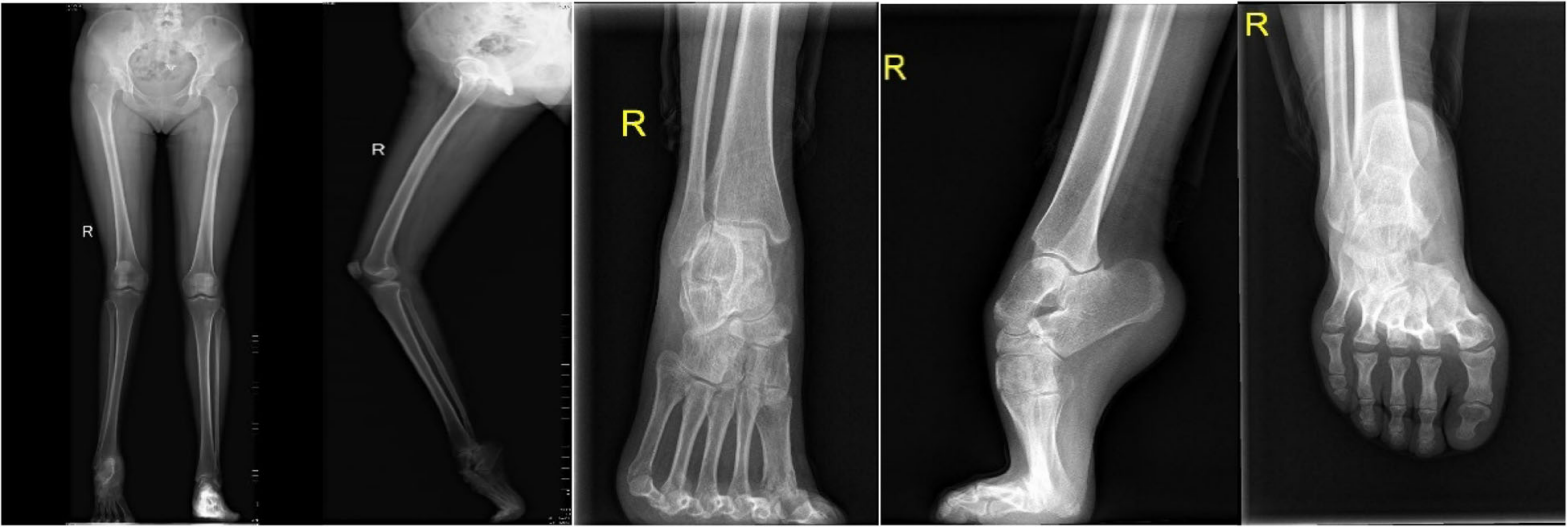
Preoperative radiological examination of the foot and lower limb

A complete radiographic evaluation is important. Weight-bearing ankle and foot radiographs including AP view, lateral view, long axis view of calcaneous are essential. The full-length X-ray of both lower limbs in the standing position is also needed (Fig. 2). A weight-bearing AP view of the ankle and foot helps to assess the condition of the ankle and whether there is rotational deformity of the foot. A weight-bearing lateral view is especially helpful. The long axes of the talus and first metatarsal create Meary’s angle. A normal measurement is 0 to 5°. The Pitch angle measures the plantar aspect of the calcaneous with the weight-bearing surface. Normal measurement is around 25°, and anything over 30° should be considered a moderate deformity. Hibb’s angle is formed by the axis of the first metatarsal with the body of the calcaneous. Normal measurement is less than 45° [15]. Long axis view of calcaneous is useful to evaluate tibia, ankle, and calcaneal deformity [16]. Normal calcaneus has a valgus angle of 0 to 5°, but the line on the vertical axis of the mid-body of the calcaneus should be parallel and approximately 1 cm lateral to the mid-diaphyseal line of the tibia [17]. A full-length X-ray of both lower limbs in standing position is to assess the lower limb forceline and whether there are rotation, angle, shortening deformity. The axial deformity of the tibia is defined by the CORA (center of rotation angulation) method with the malalignment test [18]. The center of rotation of angulation is identified by locating the intersection of the proximal and distal tibial mechanical axes. The radiological examination of foot and ankle deformity combined with lower extremity deformity requires a high level, which must be completed by a senior radiologist and orthopedic surgeon.

Computed tomography (CT) and 3D reconstruction can help us understand the deformity more intuitively. Doppler ultrasonography helps to assess if there are vascular malformations of the lower extremities. EMG (electromyography) is to assess the presence of nerve damage in the lower extremities.

For all admitted patients, the Limb Lengthening and Reconstruction Society (LLRS) AIM score [19] and the Modified Dimeglio score [20, 21] were used for preoperative evaluation.

### Operative technique

In all cases, surgery was carried out under general anesthesia without neuromuscular blockage in supine position. Systemic antibiotics were given half-hour before the operation and a tourniquet was used.

#### Tibial and fibular osteotomy

After K-wire location and guide, tibial osteotomy and fibular osteotomy were to be performed using swing blade [22]. If patients had angular deformity of the lower limb, the osteotomy plane was usually at the CORA point. If the patient only had lower leg shortening, the tibial osteotomy plane was close to the metaphysis and the fibula osteotomy plane was distal.

#### Soft tissue release

We did the percutaneous achilles tendon lengthening for patients with achilles tendon contracture. Tendon transfer (e.g., posterior tibial tendon transfer, peroneus longus strengthens peroneus brevis tendon) was used in patients with imbalanced muscle strength [4]. But, tendon transfer procedure usually is performed when the distraction procedure was completed.

#### Foot osteotomy

Different osteotomy procedures [7, 23] were applied according to the type of deformities in order to recover plantigrade foot in one stage. The osteotomy surface was fixed by K-wires or plate. For patients with forefoot deformities, midfoot osteotomy may be preferred. For patients with first-ray decline, dorsal wedge osteotomy of the first metatarsal was needed. For patients with hindfoot varus or valgus, calcaneus osteotomy was required to correct the hindfoot force line.

#### Ilizarov external frame application

We placed the Ilizarov external frame on K-wires which were inserted in the above steps. The frame consists of three main parts: the tibial base frame, the heel construct, and the forefoot construct. The tibial base frame was composed of four complete rings, the most proximal ring, and two distal rings of the tibia were fixed by two crossed K-wires. The middle ring was fixed with two crossed olive wires or one K-wire and one olive wire. The osteotomy segment of the tibia was fixed by a single Schanz pin and connected to the tibial base frame. The heel construct is a half ring attached to the calcaneum by one K-wire and additional two half pins. The forefoot part of the frame usually consists of a half ring attached to two crossed K-wires inserted transversely through the metatarsal shafts. All rings were connected by threaded rods. Appropriate hinges were attached between the three constructs to drive the deformity correction.

**Fig. 3:**
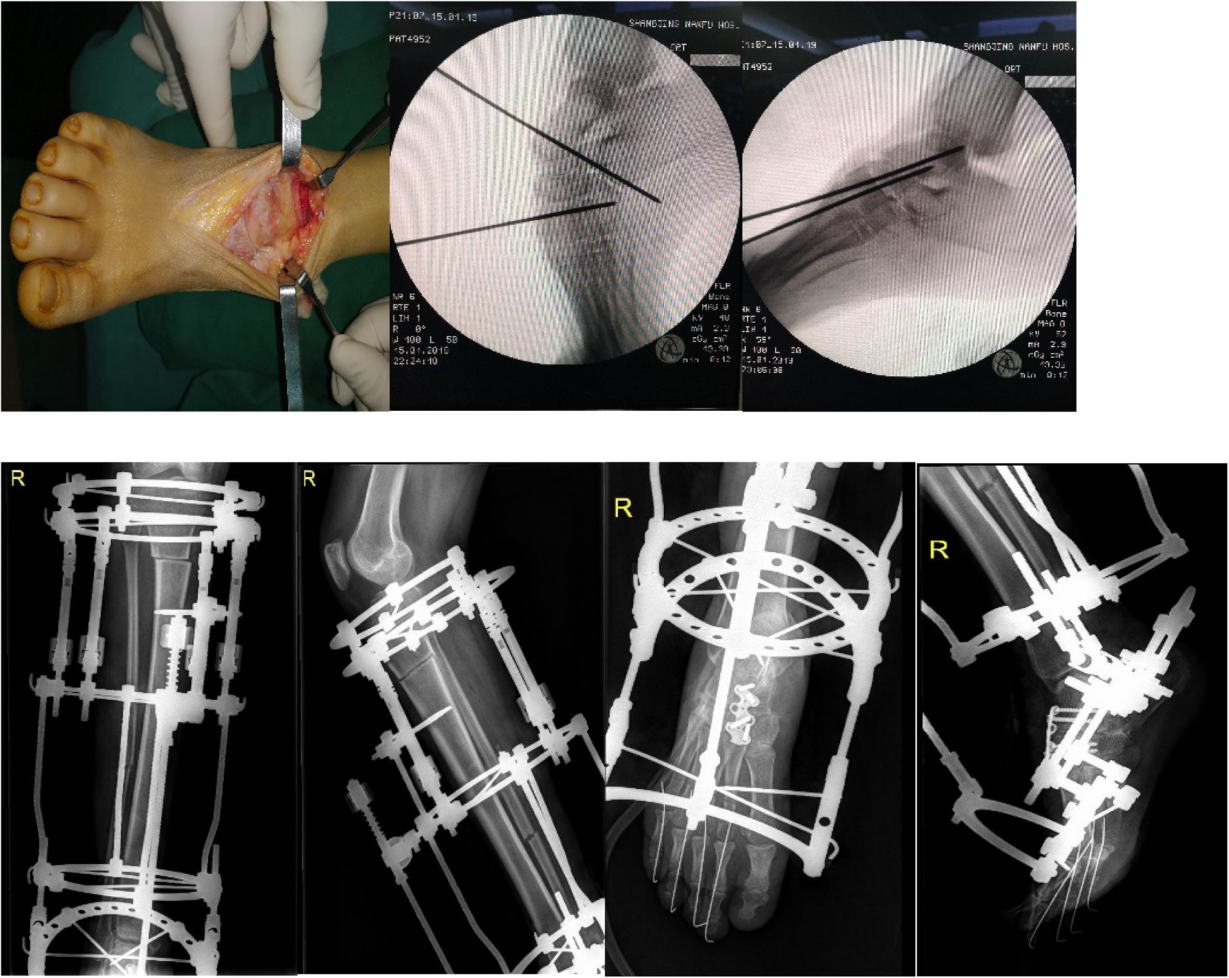
Midfoot osteotomy was done and Ilizarov external frame application

To prevent toe contracture, K-wires were inserted into all toes (Fig. 3).

C-arm fluoroscopy was used throughout the procedure to ensure ideal positioning of the fixator.

The surgical procedure is shown in Table 2.

**Table 2:** Procedure data

Osteotomy type	*n*	Soft tissue procedure	*n*
Midfoot	28	PTT transfer	14
The first metatarsal	16	ATT transfer	18
Calcaneus	20	PL transfer	32
Tibial osteotomy	32	Achilles tendon	32
Fibular osteotomy	32	lengthening	
Supramalleolar	5	Flexor tendon lengthening	10

*PTT* Posterior tibial tendon, *ATT* Anterior tibial tendon, *PL* Peroneus longus

### Postoperative care

**Fig. 4:**
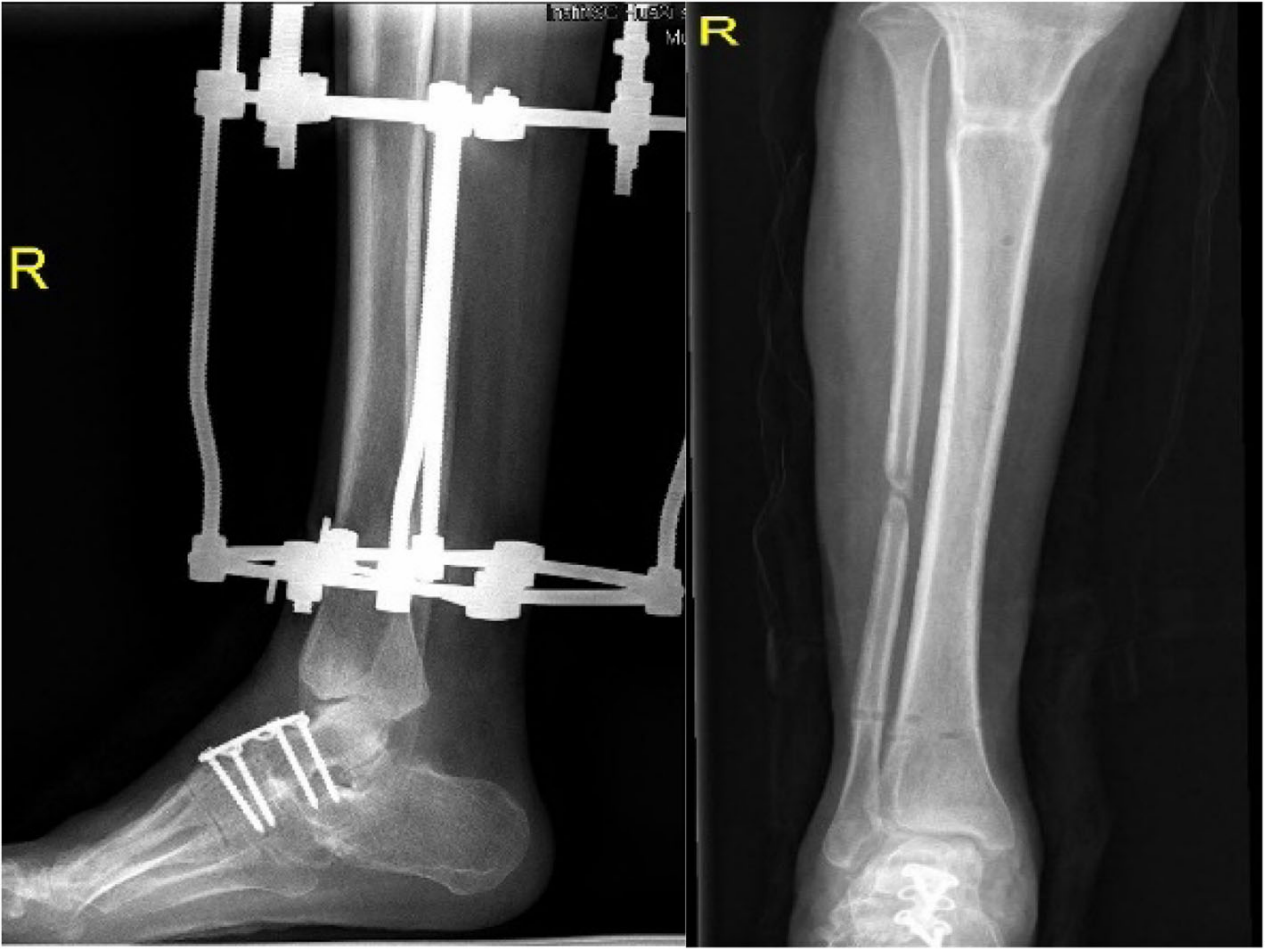
The heel construct and the forefoot construct were removed after 2 months. The tibial base frame was removed when the lower limb deformity was corrected

Correction began after a latency period of 7 to 10 days [22, 24]. Distraction was initiated in a rate of 1 mm/day, divided into four or six time intervals. In the period of distraction, the tension over the soft tissues, the neurovascular status, the pin site situation, and presence of pain were monitored regularly. Patients were encouraged to weight-bearing with the help of a walker or crutches to stimulate osteogenesis. Radiographs were taken every 2 weeks to observe how the distraction was progressing. The distraction speed and plan were adjusted based on the radiographic results. The heel construct and the forefoot construct were removed after 2 months. After full correction of the lower limb was achieved, the tibial frame remained stable for 6 weeks (Fig. 4). During this period of distraction, patients were encouraged to full weight-bearing and improvement of the gait by walking exercise.

**Fig. 5:**
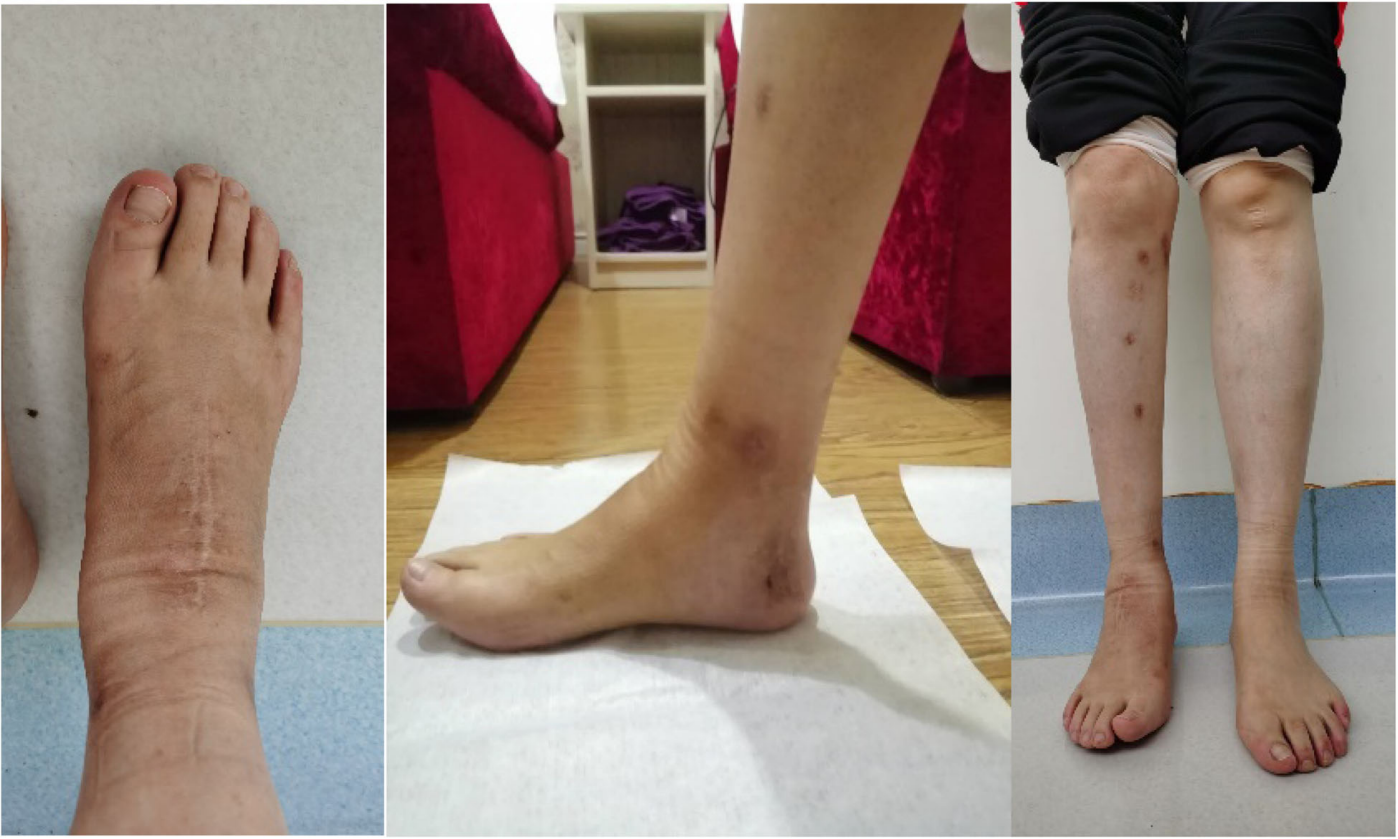
Postoperative standing appearance of the patient

After deformity correction, the Ilizarov external fixation was removed (Fig. 5). To maintain the correction and avoid early recurrence, an ankle–foot orthosis was routinely used in all patients for the following 6 months to 1 year [25, 26]. The foot component was removed 4 weeks after the surgery. Before removal, the hinge was unlocked in the daytime for range of motion exercise. At night, the hinge was locked with the ankle placed at 5–10° dorsiflexion.

During the follow-up, the Association for the Study and Application of Methods of Ilizarov (ASAMI)–Paley score was performed to evaluate bone healing and functional recovery of the limb. The modified Dimeglio score was used to evaluate the improvement of foot deformity postoperative.

### Statistical analysis

Data were expressed as means. Statistical computation of data was performed using the statistical package SPSS 22.0 (SPSS, Chicago, IL, USA). Differences between pre- and postoperation were tested by independent sample *t* test and the *P* value < 0.05 was considered statistically significant.

## Results

The mean follow-up time was 42.8 months. The mean external fixation time (EFT) was 6.5 months. The mean healing index (HI) was 1.7 months/cm. Preoperatively, 10 of the 32 patients had an average varus deformity of 8.9° (3–15°) at the proximal tibia which was corrected with asymmetrical distraction during tibial lengthening. Axial rotational deformity of the tibia was corrected at the final stage of tibial lengthening with derotation of the Ilizarov frame distal to the corticotomy site or derotation osteotomy. The external rotation of the tibia was 28° (17–45°) before operation and 5° (0–10°) after operation. All affected limbs returned to normal length with average extension of 2.9 cm (2.0–4.3 cm). Meary’s angle, Hibb’s angle, and Pitch angle were all corrected to normal. No recurrence of the deformity occurred. The mean LLRS AIM score was improved from 7.5 to 0.3. At the final follow-up, the ASAMI-Paley score was graded as excellent in all limbs in the aspect of bone results, and functional results were defined as excellent in 29 (90.6%) limbs and good in 3 (9.4%) limbs. The mean modified Dimeglio score was significantly improved from 7.2 to 1.3 (Table 3).

**Table 3:** Total results

	Preoperative	Postoperative	*P* value	
The LLRS AIM score	7.5 ± 2.2	0.3 ± 0.2	*P* < 0.05	
The modified Dimeglio score	7.2 ± 1.4	1.3 ± 0.5	*P* < 0.05	
	Bone results		Functional results	
The ASAMI-Paley score	Excellent	100%	Excellent	90.6%
	Good		Good	9.4%
	Fair		Fair	
	Poor		Poor	

Althought patients and caregivers were instructed about pin-site care, there were still ten patients with pin-site infection. After oral antibiotics and dressing changes, pin-tract infection were all controlled. Residual deformity in two patients were corrected by continued external frame adjusting around 2 weeks. Delayed healing of tibial osteotomy occurred in three patients and healed after local injection of PRP (platelet-rich plasma) or bone graft.

## Discussion

Coexistence of LLD with CFD, especially in the patients suffering unilateral congenital clubfoot, as revealed by previous studies, is not an uncommon situation. For example, Shimode reported an average limb length discrepancy of 14.6 mm [10], while Wynne-Davies reported 25 mm [27] and Little reported 21 mm [28]. Since the average age of the patients in Shimode’s study was younger than that of the latter two, it is reasonable to believe that limb length discrepancy would not improve with the patient’s growth. On the contrary, the discrepancy seems to be even more severe in adults, usually over 2 cm on average. Data from previous studies also showed that there was no statistical difference concerning limb length discrepancy in complex patients between males and females, meaning that both men and women are equally threatened. Moreover, the discrepancy is not just restricted to length. As a matter of fact, there is usually a generalized decrease in limb size by means of limb girth and foot length due to calf atrophy and poor ossification of the bones, probably because of the reduced blood supply of the limb [10]. Besides discrepancy, complex foot deformity is usually accompanied with lateral rotation of the leg, for internal and external rotation of the talo-tibiofibular unit is in coupling with inversion and eversion of the calcaneopedal unit, according to the “lamina pedis” theory proposed by I Ghanem et al. [29]. There is often coexisting angulation of the limb as a compensation of the mal-aligned hind foot as well. Therefore, clubfoot deformity and the coexisting ipsilateral lower limb deformity are disorders that share the same etiologic cause that may date back from the patient’s childhood. Together, they impair the weight-bearing function of the limb and shift the load to the opposite side. Hence, they should be regarded as a whole during the treatment.

To our knowledge, the current study is the first one that raises the concept of integrative therapy of CFD and the associated LLD as a whole. Instant correction of the CFD is achieved with necessary soft tissue release, osteotomies, and arthrodesis. The foot component of the Ilizarov frame can help correct the equinus deformity and provide some fine adjustment of the correction of the foot deformity. Moreover, with its protection over the site of midfoot osteotomy, early weight-bearing can be allowed to promote bone healing, both in the foot and the limb. However, prolonged trans-articular frame fixation may result in stiffness of the joints in the foot [30]. Therefore, we chose to unlock the hinge during daytime for range of motion exercise and remove it as early as we can. The associated lower limb deformity is corrected with gradual Ilizarov distraction procedure after the surgery. After the distraction procedure is complete, secondary tendon transfer is carried out to maintain the plantigrade foot without any influence of the distraction force that result from the Ilizarov procedure. As can be seen from the result of the current study, satisfactory correction of both the CFD and the associated LLD with relatively low risk of nonunion, neurovascular and wound complication, can be achieved via our therapeutic strategy.

However, the correction of CFD combined with LLD by using the Ilizarov procedure together with osteotomy and soft tissue release is challenging for inexperienced orthopedic surgeon. Therefore, before starting treatment of any complex deformity, it is important to grasp the patient’s expectation. Surgeons should give a realistic explanation of what the deformity correction will accomplish, what the foot will be like in the corrected position, and the functional limitations.

The current study still has limitations. First of all, the retrospective design with no valid control group has brought high risk of bias. Second, the sample size is low. Third, the follow-up period is relatively short. Therefore, a large scale randomized control study with long term follow-up is needed to prove the effectiveness and safety of the strategy proposed by the current study.

## Conclusion

Treatment of complex foot deformity combined with lower limb deformity by using the Ilizarov technique together with osteotomy and soft tissue procedure usually achieves good results. However, this method has a long learning curve. Many details still need to be continuously improved in practice.

## Data Availability

All data generated or analyzed during this study are included in this published article and its supplementary information files.

## Abbreviations

CFD: Complex foot deformities
LLD: Lower limb deformities
CTEV: Congenital talipes equinovarus
TCS: Tethered cord syndrome
CORA: Center of rotation angulation
LLRS: The limb lengthening and reconstruction society
EFT: External fixation time
HI: Healing index
PRP: Platelet-rich plasma
